# Developing a Conversational Agent’s Capability to Identify Structural Wrongness in Arguments Based on Toulmin’s Model of Arguments

**DOI:** 10.3389/frai.2021.645516

**Published:** 2021-11-30

**Authors:** Behzad Mirzababaei, Viktoria Pammer-Schindler

**Affiliations:** ^1^ Know-Center GmbH, Graz, Austria; ^2^ Institute for Interactive Systems and Data Science, Graz University of Technology, Graz, Austria

**Keywords:** Toulmin’s model of argument, argument mining, argument quality detection, educational technology, educational conversational agent

## Abstract

This article discusses the usefulness of Toulmin’s model of arguments as structuring an assessment of different types of wrongness in an argument. We discuss the usability of the model within a conversational agent that aims to support users to develop a good argument. Within the article, we present a study and the development of classifiers that identify the existence of structural components in a good argument, namely a claim, a warrant (underlying understanding), and evidence. Based on a dataset (three sub-datasets with 100, 1,026, 211 responses in each) in which users argue about the intelligence or non-intelligence of entities, we have developed classifiers for these components: The existence and direction (positive/negative) of claims can be detected a weighted average F1 score over all classes (positive/negative/unknown) of 0.91. The existence of a warrant (with warrant/without warrant) can be detected with a weighted F1 score over all classes of 0.88. The existence of evidence (with evidence/without evidence) can be detected with a weighted average F1 score of 0.80. We argue that these scores are high enough to be of use within a conditional dialogue structure based on Bloom’s taxonomy of learning; and show by argument an example conditional dialogue structure that allows us to conduct coherent learning conversations. While in our described experiments, we show how Toulmin’s model of arguments can be used to identify structural problems with argumentation, we also discuss how Toulmin’s model of arguments could be used in conjunction with content-wise assessment of the correctness especially of the evidence component to identify more complex types of wrongness in arguments, where argument components are not well aligned. Owing to having progress in argument mining and conversational agents, the next challenges could be the developing agents that support learning argumentation. These agents could identify more complex type of wrongness in arguments that result from wrong connections between argumentation components.

## 1 Introduction

Imagine an intelligent entity with whom a learner can discuss definitions of core concepts in a learning domain, moving from simply checking back whether the learner’s memory of concepts and abstract understanding of concepts is correct, toward discussing increasingly complex application concepts. This, in a nutshell, is what good teachers do; and much research in artificial intelligence for education has gone into developing computational systems that are able to, at least partially, fulfill some of the functions that (good) human tutors take on (Koedinger et al., 1997; Gertner and Kurt, 2000).

In the above description, the tutoring conversation(s) first focuses on reviewing knowledge, and then increasingly on comprehension and application of knowledge to concrete examples. Such a procedure follows the revised version (Anderson et al., 2001) of Bloom and others, (1956)’s taxonomy. Bloom’s taxonomy is a hierarchical categorization of educational goals. Essentially, it proposes to describe in which different ways one can know and learn about a learning subject. Additionally, it proposes a hierarchy in the sense of stating which steps need to be taken before others. This makes it suitable to design an intelligent tutor, in the sense of providing the intelligent tutor with a didactical structure along which to proceed. In this taxonomy, remembering, understanding, and applying are proposed as the first three types of learning with respect to knowledge that should be learned. This taxonomy hence can be understood as the design rationale for our conversational agent’s dialogue structure.

In our overarching research, we are working on a conversational agent with whom one can discuss what intelligence is, and in what sense specific entities that can be encountered in real life are intelligent or not. The choice of topic—discussing in what sense an entity is intelligent—has been made on the background of understanding the development of AI literacy as important in a society pervaded by increasingly powerful technology that is based on data analytics and other methods from AI (Long and Magerko, 2020). One puzzle piece in this is to understand what AI is (ibid); as a precursor and surrounding discussion, we see the question of what is meant by intelligence, and more specifically in what sense different entities can be understood as intelligent.

In this article, we focus on the part of the tutorial conversation where the student is asked to apply agreed-upon definitions of intelligence to a concrete (type of) entity, such as “a cat,” “a chair,” or “a self-driving car.” In the ensuing discussion, the conversational agent has the role of a tutor who develops the student’s argumentation into a reasonable and clear argument. Such an agent needs to assess both structure and content of the argument.

For assessing content-wise correctness of unconstrained answers in conversational educational answers, approaches such as comparing user responses with predefined correct answers exist (Graesser et al., 2000; Cai et al., 2020). In parallel, research on argument mining has worked on identifying argumentative parts in longer texts (Moens et al., 2007; Stab and Gurevych, 2014, 2017). In complement to such prior research, this work addresses the challenge to understand the structure of a given argument, in order for a (conversational) intelligent tutor to give specific feedback on what elements of an argument are missing. To achieve this goal, we investigate the suitability of Toulmin’s model of what components a reasonable argument has and should have (Toulmin, 2003) as a conceptual basis for a computational model of argument quality. This model has already been used differently, for example, in the field of computational linguistics (Habernal and Gurevych, 2017), outside the field (Simosi, 2003), and computer-supported learning (Erduran et al., 2004; Stegmann et al., 2012; Garcia-Mila et al., 2013).

The goal of argumentative conversational agents could be persuading the users toward a specific topic or idea (Toniuc and Groza, 2017; Chalaguine and Anthony, 2020) or just conveying the information by offering arguments that keep the dialogue comprehensive and meaningful (Le et al., 2018; Rakshit et al., 2019). These two goals do not focus on the educational aspect of argumentation.

One aspect that is missing here is the user’s argumentation or how they learn to argue. Our focus in this work is on analyzing and giving feedback on the argument structure of the human user which is novel in comparison with other works that emphasize the retrieval of suitable (counter-) arguments within a conversation over feedback or persuading users. Furthermore, in this work, we used Toulmin’s model in an educational conversational agent to teach how to argue and how a good argument should look like.

This article is organized as follows. In *Background Knowledge and Related Work*, we review ongoing research on chatbots, especially in the field of education; ongoing research on argumentation mining, and lay out Toulmin’s model of argument as background for our work. In *Research Questions*, we concretize the research questions that we ask and answer in this work. In *Methodology*, we describe the method used to answer the research questions, including data collection, annotation, inter-rater agreement, data preprocessing, feature selection, overarching model development, and evaluation process. In *Results* we describe results in line with the research questions from *Research Questions*, and we conclude the article with a discussion and conclusion *Discussion and Conclusion*.

## 2 Background Knowledge and Related Work

### 2.1 Toulmin’s Model of Argument

**FIGURE 1:**
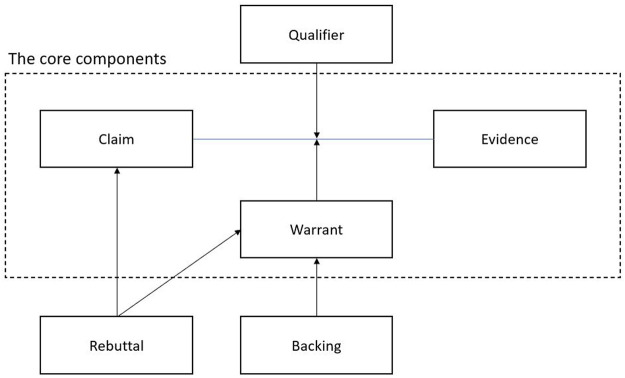
The component of arguments based on Toulmin’s scheme (Toulmin, 2003).

In this work, for argument classification or identifying the components of arguments, we used Toulmin’s model (2003) of argument. Toulmin’s model, which comes from a philosophical view, is essentially a structure for analyzing arguments. Based on Toulmin’s conceptual schema, an argument can be broken into six different components: a claim, evidence/data/observation/fact/ground, a warrant, qualifiers, rebuttal, and backing. A claim is a conclusion whose validity must be proven. Evidence is a statement or a piece of knowledge that uses to prove the claim. The connection between the claim and the evidence is established by a warrant. A qualifier is a word or a phrase that shows the certainty of the claim, for instance, “probably” or “completely.” Rebuttal can be considered as another valid view to the claim. And finally, backing refers to a cover for the warrant, especially when the warrant was mentioned implicitly. Toulmin’s components contain six different parts but based on the model, the main components are the claim, warrant, and fact or evidence (Toulmin, 2003). In Figure 1, the relation among the component is illustrated. The warrant is used as a connector between the claim and the evidence. In addition, the rebuttal and backing are considered as a cover for the claim and the warrant respectively.

Toulmin’s model of argument has also been used successfully in an educational context. In Simon (2008), the author enriched teachers in the teaching and evaluation of argumentation in science contexts by using a program by which the teachers learn how to identify Toulmin’s components in discussions and also teach students how to argue. In the program, the teachers identified the components of arguments in a list of arguments. The author indicated that using Toulmin’s model of arguments as a methodological framework could be useful for analyzing argumentation in classrooms. Toulmin’s model also has been used in computational argumentation. For instance, Habernal and Gurevych (2017) used machine learning approaches to identify Toulmin’s components of arguments in essays.

In this work, we focused on identifying the core components, claims, warrants, and evidence. This investigation has been done based on the context of a conversational agent with whom one can discuss the concept of intelligence. Conceptually, similar conversations can be carried out with respect to other concepts than “intelligence.”

### 2.2 Conversational Agents

Conversational agents are now studied in different application domains, such as for administering surveys (Kim et al., 2019), for healthcare (Müller et al., 2019), and of course, for learning (*Conversational Agents in Education*), and argumentation support (*Conversational Agents in Argumentation*).

Technically, conversational agents can be classified into two different types, retrieval and generative agents. In retrieval agents, for each turn in a conversation, a list of possible responses is considered and the most appropriate response is selected by different techniques such as information retrieval or different kinds of similarities (Le et al., 2018; Rakshit et al., 2019). Subsequently, such conversational agents rely on predefined conditional dialogue structures, where they only have the freedom to decide between different branches (notwithstanding the potential complexity, and even possible continual growth of the dialogue structure). Generative conversational agents generate the responses from scratch. The process of generating the responses is done simultaneously. The responses can be generated by different approaches. For instance, in Cahn (2017) a parallel corpus from an argumentative dialogue has been used to train a statistical machine translation by which users’ utterances have been translated to chatbot’s responses. The work we present here, as does most other related work on conversational agents in education and argumentation (see below), falls in the category of retrieval chatbots.

#### 2.2.1 Conversational Agents in Education

The vision of computational systems that resemble human tutors is old, and substantial research has and is being carried out on intelligent tutoring systems in many forms (Emran and Shaalan, 2014; Hussain et al., 2019; Montenegro et al., 2019). As both machine learning in general, and natural language processing technologies in particular, progress, conversational interaction with intelligent tutoring systems has increasingly come into focus (Io and Lee, 2017). Expectations toward conversational agents as supporting learning are high, with typical expectations on conversational agents that they address students’ sociocultural needs and engender high engagement and motivation (cp. Veletsianos and Russell, 2014) due to their offering interaction in natural language, and thereby giving an increased sense of talking to a social other.

In educational conversation, usually retrieval-based conversational agents, which have a limited set of responses, have been used. For example, in Graesser et al. (2000), for each question that their agent asks, there is a list of expectations or goals, good answers, and bad answers. The agent uses the latent semantic analysis (LSA) algorithm to calculate the similarity of users’ responses to the list of good and bad answers. LSA is a natural language processing technique for analyzing the relations between the words of documents and the different topics of documents. In Wolfbauer et al. (2020), which is a reflective conversational agent for apprentices, the responses of apprentices are matched to different concepts and then the agent’s response is selected from the poll of responses related to the concept.

In retrieval-based conversational agents, users’ responses are analyzed typically based on content and language markers. The analysis of content can be based on lexicon or patterns, for instance, Wolfbauer et al. (2020) used a dictionary-based approach and regular expressions to classify apprentices’ utterances. Also, Graesser et al. (2000) analyzed the users’ messages by language modules in which there was a large lexicon (about 10,000 words). Each entry of the lexicon contained a word with alternative syntactic classes and also its frequency of usage in the English language. In addition to the lexicon, Graesser et al. (2000) classified the learners’ content into five different classes, WH-questions, YES/NO questions, Assertion, Directive, and Short responses. The chatbot A.L.I.C.E. used AIML, Artificial Intelligence Markup Language, to match responses to different categories and then found the most appropriate response to a user input.

The subjects of the dialogues or the topics that educational agents focus on them can be various, such as mathematics (Melis and Siekmann, 2004; Sabo et al., 2013; Aguiar et al., 2014; Zhang and Jia, 2017), physics (VanLehn et al., 2002; Pérez-Marín and Boza, 2013), medicine (Frize and Frasson, 2000; Suebnukarn and Peter, 2004; Martin et al., 2009), computer science (Wallace, 1995; Weerasinghe and Mitrovic, 2011; Koedinger et al., 2013; Wang et al., 2015). In these examples, conversational agents are used to support learning about a particular subject, and the key element in all these agents is their domain knowledge (implemented by different means).

The agents also communicate with various ranges of learners for instance K-12 students, which include pupils from kindergarten to 12th grade (Wallace, 1995; Dzikovska et al., 2010), university students (VanLehn et al., 2002; Suebnukarn and Peter, 2004; Weerasinghe and Mitrovic, 2011), or apprentices (Wolfbauer et al., 2020).

In Graesser et al. (1999, 2001, 2000), a conversational agent called AutoTutor is studied. AutoTutor aims to support college students learning basic knowledge in computer science such as hardware or operating systems. AutoTutor has redefined expectations for users’ responses. It uses LSA to match the students’ responses to the expectations, and depending on which expectation is met (to which predefined answer the student response is most similar using LSA), AutoTutor selects the next step of conversation.

In the present work, similar to AutoTutor (Graesser et al., 1999; 2001; 2000), we have some expectations that we are looking for in users’ answers. However, our focus is not on analyzing and teaching content, but rather on analyzing and giving feedback on the argument structure. We also, instead of LSA or using similarity, used some classifiers to predict the next step in the conversation. This is novel w.r.t. the above-discussed literature that focuses on teaching content (Aguiar et al., 2014; Zhang and Jia, 2017), or the overall structure of a reflective tutoring conversation (Wolfbauer et al., 2020). Of course, in any fully realized conversational agent, both elements (capability to analyze and react to structure; capability to analyze and react to content) must be present.

#### 2.2.2 Conversational Agents in Argumentation

Beyond education, conversational agents have also been studied as discussion partners for general argumentation. In this, the conversational agent does not have an educational goal; having an argumentative dialogue is the goal (Le et al., 2018; Rakshit et al., 2019). For instance, in Rakshit et al. (2019), a retrieval-based agent, named Debbie, has been presented. Their agent talked to its audiences about three topics: the death penalty, gun control, gay marriage. The main goal of the agent was to keep the meaningful conversation going until it would be ended by users.

Besides the mentioned work, Chalaguine and Anthony (2020) created a conversational agent that tried to persuade its audiences regarding a specific topic, meat consumption. The agent selected an argument from its knowledge base which related to the audience’s concerns to increase the chance of persuasion. The knowledge of the agent, which was collected by a crowdsourcing method, was a list of arguments and counterarguments about the topic.

Other conversational agents tried to give information or persuade audiences about different controversial topics such as global warming (Toniuc and Groza, 2017) in which the agent had a conversation about climate change and explained the issues related to global warming.

In general, the available argumentative conversational agents can be persuasive (Toniuc and Groza, 2017; Chalaguine and Anthony, 2020) or just convey the information by offering arguments that keep the dialogue comprehensive and meaningful (Le et al., 2018; Rakshit et al., 2019).

One aspect that is missing here is the user’s argumentation or how they argue. Our focus in this work is on analyzing and giving feedback on the argument structure of the human user which is novel w.r.t. the above-discussed literature that emphasizes the retrieval of suitable (counter-) arguments within a conversation over feedback. Again, of course, in any fully realized educational conversational agents, both elements (specific feedback on learner’s argument structure, and presentation of similar or different arguments as a basis for further developing an argument content-wise) must be present.

### 2.3 Argument Mining

Argument mining or argumentation mining is a subfield or research area in natural language processing. Basically, it refers to the automatic identification and understating of arguments in a text by machines. It is one of the challenging tasks in natural language processing. Based on Wambsganss et al. (2020), there are three different levels in argument mining, argument identification, discourse analysis, and argument classification. The machine learning approaches applied for these levels could be supervised, which needed an annotated dataset, or unsupervised, which eliminated the need for annotated data. In the rest of the section, we, first, focus on the supervised learning approaches and then unsupervised learning approaches.

In the first level, identification argument, the main goal is extracting or detecting the parts of documents that contain an argument; in other words, the parts are classified into argumentative and nonargumentative (Moens et al., 2007; Stab and Gurevych, 2014; Poudyal et al., 2016; Zhang et al., 2016). For instance, in Zhang et al. (2016), the main research goal was to design a model that can detect argumentative sentences in online discussions. However, in Poudyal et al. (2016), they focused on case laws that in terms of formality are completely different from the online discussions. In another research, Dusmanu et al. (2017) tackled the first level of argument mining by identifying argumentative sentences in tweets. After detecting argumentative sentences, they classified them as factual information or opinions with using supervised classifiers. Finally, the source of factual information, which was extracted in the previous step, was identified.

The second level of argument mining is discourse analysis which refers to identify the relations, as for support or an attack, among the claims and premises in documents (Palau and Moens, 2009; Cabrio and Villata, 2013; Boltužić and Šnajder, 2014). Similar to Zhang et al. (2016), in Boltužić and Šnajder (2014), the authors dealt with online discussions. They tried to match users’ comments to a predefined set of topics, which can be either supported or not supported.

In the last level, argument classification refers to classify the components of arguments (Mochales and Moens, 2011; Rooney et al., 2012; Stab and Gurevych, 2014). In this case, argumentative parts can be classified into different classes, such as claims and premises (Mochales and Moens, 2011; Rooney et al., 2012; Stab and Gurevych, 2014) or claim, backing, rebuttal, premise, and refutation based on Toulmin’s (2003) model of argument (Habernal and Gurevych, 2017). For example, Habernal and Gurevych (2017) proposed a sequence labeling approach in which many different types of features such as lexical, structural, morphological, semantic, and embedding features were used to vectorize sentences. The authors used SVM^hmm^ (Joachims et al., 2009) which is an implementation of Support Vector Machines specifically used for sequence labeling^1^. The authors annotated the documents based on the BIO encoding. This encoding is used to distinguish which is the minimal encoding for distinguishing the boundary of argumentative components, and works as follows: The first word of an argumentative component is labeled with *B* which means the beginning of component. The label *I* is used for the rest of the words in the component. All tokens in nonargumentative components are labeled with *O*.

There are also works that tackled all the levels mentioned by Wambsganss et al. (2020). In Wang et al. (2020), the authors deal with online discussions about usability on issue-tracking systems of open-source projects. Since a large number of issues and comments with different perspectives are posted daily, the contributors of projects face a major challenge in digesting the rich information embedded in the issue-tracking systems to determine the actual user needs and consolidate the diverse feedback. Thus, the authors’ ultimate goal was to make usability issues more readable. To do this, they first discriminated argumentative comments, then, classified the argumentative comments based on two independent dimensions, components and standpoints.

Technologically, a range of classical methods of machine learning has been applied to address different levels in argument mining. For instance, in Moens et al. (2007), multinomial naive bayes classifier and maximum entropy model were used as classifiers for detecting arguments in legal texts. They converted the sentences to feature vectors which contained unigrams, bigram, trigrams, verbs, argumentative keywords such as “but,” “consequently,” and “because of,” statistical features namely average of word length and a number of punctuation marks. In Goudas et al. (2014), the authors studied the applicability of some machine learning classifiers on social media text in two steps. First, they identified argumentative sentences by using different machine learning techniques such as Logistic Regression, Random Forest, and Support Vector Machine and second, through using Conditional Random Fields, the boundary of the premises in argumentative sentences was detected. Other machine learning methods such as support vector machine (Rooney et al., 2012; Sardianos et al., 2015; Habernal and Gurevych, 2017), logistic regression (Levy et al., 2014; Rinott et al., 2015; Dusmanu et al., 2017), random forest (Eckle-Kohler et al., 2015; Dusmanu et al., 2017), and conditional random field (Goudas et al., 2014; Sardianos et al., 2015) are also used in argument mining.

In Wambsganss et al. (2020), an adaptive tool, named AL, by which students received feedback on the argumentative structure of their written text, was designed, built, and evaluated. They tried to answer two research questions that were about the acceptance of AL and also how much it was effective for users to write more persuasive texts. For the latter research question, first, they created two different classifiers by which they identified argumentative sentences and also the relation among them, supported and non-supported. Second, they evaluated the texts by measuring readability, coherence, and persuasiveness. By illustrating these scores and their definitions, users understood how to improve their texts.

In Shnarch et al. (2017), they presented an algorithm, named GrASP (Greedy Augmented Sequential Patterns), which was weak labeling of argumentative components using multilayer patterns. The algorithm produced highly indicative and expressive patterns by augmenting input n-grams with various layers of attributes, such as name entity recognition, domain knowledge, hypernyms. By considering many aspects of each n-gram, GrASP could identify the most distinguishing attributes and also iteratively extended the extracted patterns by using the information from different attributes. The greedy part of the algorithm was related to the end of each iteration in which the top k predictive patterns were kept for the next iteration.

Besides the supervised machine learning approaches that rely on annotated training data, there are unsupervised approaches that eliminated the need for training data. For instance, Persing and Ng (2020) developed a novel unsupervised approach that focused on the task of end-to-end argument mining in persuasive student essays collected and annotated by Stab and Gurevych (2017). They applied a bootstrapping method from a small dataset of arguments. They used reliable contextual cues and some simple heuristics, which relied on the number of paragraphs, the location of the sentence, and the context n-grams, for labeling the different components of arguments.

Another unsupervised approach has been presented by Ferrara et al. (2017). Their approach was based on the topic modeling technique. In their research, they focused on detecting argument units that were at sentence-level granularity. Their method, named Attraction to Topics (A2T), had two main steps. The first step was identifying the argumentative sentences and the second step was classifying the argumentative sentences, which were discovered in the first step, to their role, as major claims or the main standpoint, claims, and premises.

In comparison with the previous works and the mentioned literature, in this article, we worked on an argumentative-educational conversational agent in which the agent gave feedback on missing core components. The conversational agent tried to teach argumentation instead of persuading users or giving (counter-) arguments based on similarity to continue the conversations. The challenge we address in this article is identifying the core components of arguments based on Toulmin’s model of arguments (Toulmin, 2003), namely claim, warrant, and evidence or grounds. Others have already identified these elements based on traditional machine learning algorithms such as Random Forests (Breiman, 2001) and SVM (Joachims, 1998). In line with these authors, also in our work we use traditional ML methods such as K-Nearest Neighbors, SVM, Decision Trees, Random Forest and Ada Boost. We explicitly do not use deep learning methods in this work, as we have too little data; and do not use transfer learning as no suitable models from sufficiently similar problems are available.

## 3 Research Questions

In pursuing our overall goal, to study how to enable a conversational agent to identify different types of structural wrongness in an argument, we here investigate the suitability of using Toulmin’s model of argument within conversational agents to operationalize what a good structure of an argument is, and subsequently to identify different structural wrongness. In the present article, we study a conversational agent with whom one can discuss a single question: Is < an entity > intelligent, and in what sense? In this domain of discussion, we ask and answer the following three research questions:

• RQ1 (overarching): Can Toulmin’s model of argument be used to model different types of structural wrongness within conversational agents in the given domain?
• RQ2: How well can components of Toulmin’s model of argument be identified in the given domain?
• RQ3: Can a conditional dialogue structure with conditions based on the existence of components from Toulmin’s model of argument lead to coherent conversations in the given domain?

Our methodology is as follows:

• Develop classifiers that operationalize Toulmin’s model of argument to provide evidence for RQ2 (how well can different elements of Toulmin’s model of argument be identified) in this case (preparatory work: *Apparatus—Educational Scenario “Is < an entity > Intelligent or Not? Why?”, Data Annotation, Inter-rater Agreement, Data Processing, and Feature Selection*; classifier development and evaluation in *Results*)
• To set up a conditional dialogue structure with conditions based on the existence of arguments following Toulmin’s model of argument and show, by example, that it can lead to a coherent conversation (existential proof by example; in answer to RQ3).
• To discuss the overall suitability of Toulmin’s model of argument as a suitable basis for modeling different types of wrongness in conversational agents (RQ1) based on results on the collected dataset.

As discussed in the related work and background section above, by answering these research questions, we contribute to the existing scientific discourse around conversational agents in education and argumentation mining knowledge about how Toulmin’s model of argument can be operationalized and how this operationalization can be used within a conversational agent to allow a coherent conversation that helps users develop a—structurally—good argument. This is useful, and novel in complement to existing research and knowledge on conversational agents that use domain background knowledge to facilitate the acquisition of factual knowledge, to develop argumentation along the dimension of content or educational conversational agents that moderate discussions by injecting discussion prompts.

## 4 Methodology

Below we describe the data collection study (*Data Collection*), the educational materials used in the data collection (*Apparatus—Educational Scenario “Is < an entity > Intelligent or Not? Why?”*), the data annotation process and labels used (*Data Annotation*), the achieved inter-rater agreement as a measure of the quality of annotations and subsequently datasets (*Inter-rater Agreement*), the data preprocessing (*Data Processing*), and finally the feature selection for the three classifiers that aim to identify the existence of a claim, a warrant, and evidence in a given user statement (*Feature Selection*).

### 4.1 Data Collection

To collect data, Amazon Mechanical Turk^2^ (MTurk) was used. It is a system for crowdsourcing work and has been used in many academic fields to support research. By using crowdsourcing methods, a large number of diverse arguments can be collected and the data are free from researchers’ bias (Chalaguine et al., 2019).

The data were collected in three rounds. In each round, essentially the question *“Is < an entity > intelligent or not? Why?”* was asked to study participants. The materials prepared for all three rounds are described in *Apparatus—Educational Scenario “Is < an entity > Intelligent or Not? Why?”* below.

To increase the chance of having data without spelling or grammatical errors and also meaningful errors, we defined some qualification requirements for participants. The participants, who wanted to answer the questions, were required to be *master* workers. It means they needed to have a minimum acceptance rate of 95% in order to qualify to answer the questions. This qualification requirement ensures the high quality of the results. Furthermore, an additional qualification requirement was considered which was having an academic degree equal to or higher than a US bachelor’s degree. The reason behind that was to have better responses in terms of formality and without spelling or grammatical errors. The data have been collected in three rounds (see Table 1).

**TABLE 1:** MTurk experiments for collecting data.

Datasets	Number of collected responses	Qualification requirement
Dataset 1	100	• HIT Approval Rate (%) ≥ 95
Dataset 2	1,026	• HIT Approval Rate (%) ≥ 95
		• At least US Bachelor’s Degree
Dataset 3	211	• HIT Approval Rate (%) ≥ 95
		• At least US Bachelor’s Degree

As it is shown in Table 1, in the first pilot study, 100 responses regarding the question, *“Is < an entity > intelligent or not? Why?”* have been collected and the only qualification requirement was having an approval rate of more than or equal to 95. In the second round, 1,026 responses were collected. However, the second qualification was also added. In the last round, the same qualification requirements similar to the second round were used and 211 new responses have been collected to use as a test set. In the end, overall, 1,335 records have been collected. The data that have been collected from the first two rounds, datasets 1 and 2, are considered as validation data and training data, and the records of the last round, dataset 3, are considered as test data.

### 4.2 Apparatus—Educational Scenario “Is < an entity > Intelligent or Not? Why?”

We prepared the following materials for data collection: five different definitions of intelligence with brief explanations for each definition, a list of eight entities, and defining some properties for a good response, and a few samples of good/bad responses.

The following five definitions were given and explained as follows to study participants: There are plenty of definitions by which something or someone would be called intelligent. In this task, we focus on five of them. We will call an object intelligent if it thinks humanly, acts humanly, thinks rationally, acts rationally; or if it is able to learn from experience to better reach its goals.

These definitions were chosen on the background of understanding intelligence as a foundational concept for arguing about capabilities as well as non-capabilities of artificial intelligence. The first four are discussed as having an impact on the discussion around intelligence in relation to the development of artificial intelligence and inspired different directions of artificial intelligence research (cp. Russell and Peter, 2002). The fifth definition more closely mirrors the understanding of learning in psychology and learning sciences.

Every study participant was asked to decide and argue about the intelligence of one (type of) entity, which was chosen such that in each dataset, the following categories are similarly represented: *Inanimate objects, plants, animals, AI-enabled technologies*. These categories are ontologically different, general judgments about their intelligence are possible, and we can expect different types of argumentations per category. As a general judgment, inanimate objects can be considered to not be intelligent according to any definition, plants could be with some difficulty argued to be intelligent as a species if evolutionary aspects are put to the forefront, and animals and AI-enabled technologies could, in general, be argued to be intelligent even though in a differentiated manner.

In dataset 1, these categories were instantiated by: *tables* (inanimate object), *trees* (plants), *cats, fish* (animals), and *Google search engine* (AI-enabled technologies)*.* We collected 100 records for dataset 1 (see Table 1) which means 20 records for each entity.

For datasets 2 and 3, we used two examples per category, and these were *office chairs* and *the New York Statue of Liberty* (inanimate objects), *sunflowers*, *Venus flytraps* (plants), *snakes*, *monkeys* (animals)*, self-driving cars*, *Google search engine* (AI-enabled technologies). We collected 1,000 records for dataset 2 which were 125 records for each entity and 200 records for dataset 3 which were 25 records for each entity. We collected more records for datasets 2 and 3. The extra records were due to a few short answers because we asked others to answer them again.

During collecting the data, it was also explained that a good response should be argumentative, contain a claim, reasoning, and an explanation, have at least 10 words, and be checked again for correcting typos. Furthermore, examples of good and bad responses were also illustrated in the explanation. In Table 2, some statistics related to the collected data are shown. For datasets 1, 2, and 3, we collected 20, 125, and 25 responses respectively for each entity. Since some of the responses were too short or irrelevant, we did not approve of them and then asked new participants to answer them again. That is the reason behind the small deviations in the number of responses for each category. However, we used all the responses, rejected and approved responses, in our models. Overall, 349, 332, 329, and 327 responses were collected related to animals, plants, inanimate objects, and AI-enabled technologies respectively.

**TABLE 2:** The descriptive statistics of different categories of entities in the datasets.

Category	Datasets 1 and 2		Dataset 3 (test data)	
	# of responses	The average number of tokens	# of responses	The average number of tokens
Animals	296	36.73	53	29.77
Plants	277	34.06	55	34.85
Inanimate objects	277	31.54	52	30.23
AI-enabled technologies	276	39.13	51	32.31

### 4.3 Data Annotation

The whole annotating process was done by two annotators (the authors). The whole process had three steps. Frist, in a group session, we reached a conclusion about definitions of each component and how to annotate them. Second, we randomly selected 100 records from dataset 2 and annotated them separately to measure the agreement (The detail of measuring inter-rater agreement is mentioned in the next section). In the last step, the first author annotated the rest of the unannotated data. The data were annotated based on three core components of Toulmin’s model of arguments: Claim, warrant, and evidence (cp. *Argument Mining* and Figure 1, Toulmin, 2003).

Three different annotation values were considered for the claim: “positive” that means the user claimed that the entity is intelligent; “negative” that refers to the opposite direction which means the user’s claim is that the entity is not intelligent, “unknown” refers to responses in which there is no specific claim or stance regarding the question.

For the warrant, two different values were considered, “with warrant” or “without warrant” which refers to the existence of a warrant in the response: “with warrant” is assigned to responses in which at least one of the definitions of intelligence is mentioned.

For evidence, a binary value was considered. The responses are annotated with “with evidence” if there are some parts in the responses in which users use their background knowledge or observation to justify their claims. Table 3 represents the collected data in terms of these labels. The collected and annotated data are accessible for other researchers as an appendix to this publication^3^. We explicitly discarded at this stage an evaluation of how reasonable the evidence is; this is discussed further in *Can Toulmin’s Model Of Argument Be Used To Model Different Types Of Structural Wrongness Within Conversational Agents In The Given Domain? (RQ1)*.

**TABLE 3:** The number of different labels for each component in training and test data.

Component	Claim			Warrant		Evidence	
Annotation	Positive	Negative	Unknown	With warrant	Without warrant	With evidence	Without evidence
Training data (datasets 1 and 2)	477	594	55	691	435	835	291
Test data (dataset 3)	102	99	10	111	100	159	52

### 4.4 Inter-rater Agreement

One of the reasons that make argumentation mining and its sub-tasks such a challenging task is having disagreements in annotating datasets. Most datasets that are available do not report inter-rater agreements (Lippi and Torroni, 2016). In principle, the overall quality of the argument is something that humans cannot agree about sometimes because, based on Wachsmuth et al. (2017a), some parts of the argumentation quality are subjective, and overall quality is hard to measure. Wachsmuth et al. (2017a) also showed that some dimensions of argument quality in practice were not correlated to any argument quality in theory or some practical dimensions could not be separated and matched to theoretical dimensions of argument quality.

In general, analyzing arguments and annotating texts is controversial most of the time and it leads to having more challenges in tasks such as detecting claims, warrants, or evidence. To train and evaluate the performance of detecting the core components, high-quality annotated datasets are required. In this article, Cohen’s κ value is used for evaluating inter-rater agreements. In this method, the inter-rater agreements among the labels and agreements occurring by chance are taken into account. The equation for κ is

$$\kappa =\frac{\mathrm{Pr}\left(a\right)-\mathrm{Pr}\left(e\right)}{1-\mathrm{Pr}\left(e\right)}$$

In this equation, Pr(a) is the relative observed agreement among raters, and Pr(e) is the hypothetical probability of chance agreement. Different thresholds are defined for the value of κ. In general, the range of κ is from zero to one and the higher amount means the higher agreement between the raters. If the raters are in complete agreement then κ = 1, if there is no agreement among the raters other than what would be expected by chance, κ = 0. Based on Landis and Koch (1977), the values below 0 are considered as poor, between 0 and 0.20 as slight, between 0.21 and 0.4 as fair, between 0.41 and 0.6 as moderate, between 0.61 and 0.80 as substantial, and above 0.81 as almost perfect inter-rater reliability. In Stemler and Tsai (2008) the threshold of 0.5 was recommended for exploratory research. For natural language processing (NLP) tasks, the agreement is considered as significant when κ is greater than 0.6 (Cabrio and Villata, 2018). The values of κ for the claim, warrant, and evidence components were 0.94, 0.92, and 0.65 respectively. The κ value for the claim and warrant is more than 0.9 which means there is almost perfect inter-rater reliability. The definitions of claim and warrant components are straightforward and the coders exactly know what they are looking for. In contrast, the evidence could be anything based on users’ background knowledge or observations that are related to the users’ claim. So, there is a chance that in some responses the coders have different opinions. Even though there are unlimited ways of providing evidence that supports the claim of whether and in what sense an entity is intelligent, there is substantial agreement between the two raters on the existence of evidence (κ = 0.65). In an analysis of disagreements, the disagreements mostly stemmed from different quality thresholds of raters on what would be acceptable to count as evidence or not. For instance, there were disagreements for these samples, “No. The statue of liberty cannot think and has no mind or brain.” or “An office chair is not intelligent because neither it can do work on its own nor it can think and act.”

### 4.5 Data Processing

The preprocessing steps are the same for all the models we created for detecting claims, warrants, and evidence. The steps are as follows: 1) converting all responses to lowercase form, 2) removing additional spaces in beginning, ending, and middle of the responses, 3) replacing the various form of the entities’ names with a specific token, “ENT,” 4) tokenizing and lemmatizing the responses.

Replacing the entities’ names is crucial. It is mandatory for two reasons. First, by replacing the entities’ names with “ENT,” it will be possible to create only one claim detection model to cover all types of entities. Second, we wanted to ignore the impact of the entities on the prediction because the names of entities will affect the prediction of models. For example, in 86 per cent of responses in which we asked about the intelligence of monkeys the users’ claims were positive. It means the model of detecting claim tends to assign a positive claim to the responses related to the monkey entity. This justification is also valid for other entities such as “an office chair.” In 91 per cent of responses related to office chairs, the claim was negative which means the users claimed that the entity is not intelligent. In the next subsection, the features used to create the models are presented.

### 4.6 Feature Selection

To create classifiers, user responses need to be converted to vectors in order to be used by machine learning classifiers. In this subsection, we report on features in the sense of how the vectors are created. Overall, we developed three classifiers, one for each core component of Toulmin’s model of argument: claims, warrants, and evidence (see *Argument Mining*). For each classifier, different features were used; and we report for each classifier separately which features were used below. Some features were nonetheless shared for all classifiers (general features—namely TFIDF representation of the user response), and some features were component-specific, i.e. specific to the core component of Toulmin’s model of argument.

#### 4.6.1 Claim

We report on features that were used as input to the classifiers that aimed to detect the existence of a claim (see *Argument Mining*) in user response. We aimed to differentiate between three classes, positive claims, negative claims, and unknown claims. We identified these classes with two groups of features, general features and component-specific features.

Term-frequency-inverse-document-frequency (TFIDF) was used in this work throughout as a general representation of user responses: As a full document set, datasets 1 and 2 are used; and the dictionary vector contains bigrams and trigrams. The unigrams were ignored because they could not be informative and indicative. The words such as “is,” “intelligent,” “not” did not lead us to a correct prediction about claims. However, bigrams and trigrams, for instance, “is intelligent” and “is not intelligent” are what we have needed to predict users’ claims. After preprocessing steps (see *Data Processing*), only the 500 most frequent bigrams and trigrams for the whole datasets 1 and 2 were used as TFIDF vectors. The underlying rationale was, to avoid high sparsity vectors.

In addition, we used general background knowledge as well as information from pilot studies to add features that needed to be considered both specific to the “claim” as one component in an argument that shall be classified; and that was specific to the particular question that has been asked (is an entity intelligent or not). Two regular expressions were used to indicate whether a response was started or ended by phrases or words such as “yes,” “no,” “it is intelligent,” “it is not intelligent” or not. If one of these patterns can be found in a response, based on being positive or negative, a ternary value, −1, 0, 1, was added to the general feature vector of the response.

#### 4.6.2 Warrant

In this study, we asked participants to use one of five definitions of intelligence as linking between their claim and their concrete evidence: “acting humanly,” “acting rationally,” “thinking rationally,” “thinking humanly,” and “learning from experience to better reach its goals.” We aimed to differentiate between two classes only: With a warrant in the sense of a reference to one of these definitions, and without a warrant. Note that in Toulmin’s description, it is said that a warrant can also be implicit as underlying understanding based on which a human is making an argument. In this work, we were looking for explicit warrants in labeling, and subsequently in classification.

Part of the feature vector for the warrant classifier was the same TFIDF representation of the user response (the length of vector: 500; terms, bigrams and trigrams are represented). Our goal was to identify the existence of warrants (or their absence) in the sense of detecting the usage of one of five predefined definitions of intelligence. Hence, regular expressions were used, as a component-specific feature, to detect the presence of the different forms of definitions in responses. The phrases that we looked for by regular expressions were indicative phrases such as “act humanly,” “think rationally,” “can learn,” “learn from experience,” or “reach better.” Based on the existence of these patterns, a binary value, 0 and 1, was added to the general features.

#### 4.6.3 Evidence

We aimed to differentiate between two classes, that some evidence is given, or that it is not. Since no predefined list of facts or observations was given in the present studies, the evidence part in our study was the most free and hence most variable part of user responses. As general features, similar to other components, we used TFIDF vectors but with different parameters. As a full document set, datasets 1 and 2 were used; and the dictionary vector contained unigrams and bigrams. In contrast to the claim and warrant that trigrams phrases can be indicative for identifying the existence of claims and warrants, for detecting evidence component, unigrams and bigrams, such as “no brain,” “inanimate object,” “prey,” or “making tools,” can be discriminative. Furthermore, in contrast to the feature vectors for detecting claims and warrants, the 3,000 (instead of 500) most frequent unigrams and bigrams for the whole dataset were used in the dictionary vector, and TFIDF was computed based on this reduced dictionary vector. The underlying rationale for length reduction was again to avoid high sparsity vectors; the length was still larger because we expected more reasonable variance in the evidence part of the given arguments. Besides the length of vectors and n-grams, to remove phrases related to the claims and the warrants, we ignored all phrases that occurred in more than 30 per cent of all responses to have more relevant and meaningful phrases.

As component-specific features, we used two different evidence-specific feature sets, a list of evidence-specific keywords, and the length. The evidence-specific keywords where we assume that when one of them appears in the user response, there is a high likelihood that this keyword is part of the evidence for the argument, and hence that the statement should be in the “with evidence” class. The evidence component is the only argument component in which users need to talk about aspects specific to the entities and based on their experience and background knowledge. It means that users use their own keywords to justify their claims. Table 4 shows the 30 keywords that we identified in dataset 2. To identify these keywords, we used dataset 2 and did the following preprocessing: First, all phrases related to the claim and warrant (component-specific features) were eliminated. Second, we extracted unigrams and removed stop words. For each remaining unigram, a vector with the length of the number of responses was created that showed the existence of the unigram in each response. Then, Matthew’s correlation coefficient between each vector of unigrams and the class values of the evidence class (with/without evidence) was calculated. The 500 most correlated unigrams were chosen; the cutoff was empirical because subsequent unigrams seemed too random. This yielded the bold entries in Table 4. The non-bold entries are the keywords that have been added to the list because they are synonyms, similar, or relevant to other high-correlated unigrams such as “by human,” “handmade,” and “made by” as synonyms or relevant to “man-made.”

**TABLE 4:** The 30 terms that correlate most with the class “with evidence” in dataset 2.

**Instinct**	**Plant**	**Prey**	**Steel**	**Inanimate**	**Sunlight**
Hunt	**brain**	**object**	**trap**	handmade	**living**
Survive	**lifeless**	**aware**	**insect**	**man made**	**grow**
**Tool**	alive	**cognition**	feed	made by	**food**
**group**	**metal**	**program**	by human	**stone**	**sun**

These words are related to entities and study participants often used them in the evidence part of responses to argue why a particular definition of intelligence (warrant) applied to an entity or not. This feature set conceptually captures evidence that is made at an abstraction level that is higher than the single entity types in the sense of referring to an entity’s decisive characteristic as being an inanimate object, or as having a brain; which in turn would be true about many more entities than the Statue of Liberty, or snakes. To use this feature, a binary value was added to the general vector of evidence to indicate the existence of this evidence-specific feature.

The second evidence-specific feature set was the length in terms of the number of words of the responses. Conceptually, if one wants to make a claim, refer to a predefined definition and in addition describe evidence that links claim and warrant, one needs more words than if one does not add evidence. When cross-checking this intuition in datasets 1 and 2, there is a significant difference between responses that contain evidence (M = 39.16, SD = 23.27, in datasets 1 and 2) and without evidence (M = 25.10, SD = 16.73, in datasets 1 and 2). To show this, Welch’s *t*-test experiment was done and it showed that the difference was significant, t = −11.07, *p*-value< 0.0001 and the degree of freedom = 701.63. To show that the significant difference was due to the length of the evidence component and not the other components, first, we reduced the length value by 4 and 5 words if responses had the claim and the warrant component respectively. Then, we did Welch’s *t*-test again on the new values for the length feature for responses with evidence (M = 32.27, SD = 22.9) and without evidence (M = 18.39, SD = 16.86). Based on the new length value, there was a significant difference in terms of length, t = −10.95, *p*-value< 0.0001, and the degree of freedom = 684.48. This feature intuitively makes sense, and yet of course is very coarse, in the sense that it can fail in single instances if no claim or warrant exists (shorter overall response that still includes evidence), can fail in single instances if claim and warrant are expressed very verbosely; and of course, absolutely fails to capture the correctness of the evidence or soundness of the overall argument in any way.

In Table 5, we summarized the features that were used for identifying the existence of the core components in responses.

**TABLE 5:** The features used for training classifiers of claim, warrant, and evidence components.

Component	General feature	Component-specific feature
Claim	• TFIDF of bigrams and trigrams (The length of vector = 500)	• Regular expressions to indicate phrases such as “it is (not) intelligent”
Warrant	• TFIDF of bigrams and trigrams (The length of vector = 500)	• Regular expressions to indicate the proposed definitions of intelligence
Evidence	• TFIDF of unigrams and bigrams (The length of vector = 3,000)	• The entity-specific keywords (Table 4)
		• The length of responses based on the number of words

## 5 Results

### 5.1 How Well Can Components of Toulmin’s Model of Argument Be Identified in the Given Domain? (RQ2)

In this section, we answer RQ2—How well can different elements of Toulmin’s model of argument be identified? by developing classifiers for the three core components of Toulmin’s model of argument, namely claims, warrants, and evidence (cp. *Argument Mining*) based on datasets 1 and 2 as training datasets and dataset 3 as unseen test data for evaluating these classifiers (see especially *Data Collection* on Data Collection and the three different datasets). The classifiers were developed using vector representations of user statements using features as described above (*Feature Selection*). We use traditional ML methods such as K-Nearest Neighbors, SVM, Decision Trees, Random Forest, and Ada Boost. We explicitly do not use deep learning methods in this work, as we have too little data; and do not use transfer learning as no suitable models from sufficiently similar problems are available.

For selecting the best classifier for each core component, we measured F1-score in 10-fold cross-validation on dataset 2 for mentioned traditional ML methods. Furthermore, we used dataset 1 as a held-out dataset to compare the ML models based on F1-score. After we had selected the best classifier, a final model was trained based on both datasets 1 and 2. To avoid overfitting, the dataset for tuning hyperparameters is a little bit larger than that for initial model training and comparison and more diverse (datasets 1 and 2 have been collected with slightly different materials—see *Apparatus—Educational Scenario “Is < an entity > Intelligent or Not? Why?”*); and the dataset for evaluation needed to be previously unseen, as is standard practice in ML literature. We note that datasets 1 and 2 were lexically relatively similar, first because we had removed concrete entity names in preprocessing (replacement with ENT), and second because user arguments differ mainly across categories of entities (inanimate object, plant, animal, AI-enabled technology) and not so much between different entities (e.g., cat vs. snake).

#### 5.1.1 Claim Component

In this subsection, we describe the development and evaluation of a classifier for deciding the existence and the direction of a claim. In Table 6, you can see real responses, before applying preprocessing steps, related to the different values of a claim.

**TABLE 6:** Real samples regarding the different values of the claim component. There are users’ responses without any modification.

User’s response	Claim
“Monkeys and humans are evolutionary speaking very close. Whilst it can’t be said to think or act “humanly” (by definition only humans can do that), it can certainly think and act both intelligently and rationally, and most certainly learns from experiences. Therefore it is intelligent.”	Positive
“I think that a self-driving car is intelligent. It learns from experiences and adapts and makes decisions based on what it has learned.”	Positive
“I think a venus flytrap just wants to feed itself. That would be the goal it wants to reach.”	Unknown
“the New York Statue of Liberty is made of copper and it exhibits positivity to the people around it and also the toes of this statue denotes the stableness to the world.”	Unknown
“no I don’t believe a self-driving car is intelligent I believe the people who wrote the code that make the car self-driving are intelligent. The car can only do what is it is programed to do.”	Negative
“no”	Negative

We compared standard machine learning classifiers (K-Nearest Neighbors, SVM, decision tree, Random Forest, Ada Boost) using 10-fold cross-validation over dataset 2 and evaluation of performance on dataset 1 as a held-out dataset to identify the best classification model. To compare classifiers, we report the mean and standard deviation of macro-F1 scores over all training-and-test iterations. The result is shown in Table 7.

**TABLE 7:** The result of 10-fold cross-validation on dataset 2 in detecting claims and evaluation of performance on the held-out dataset.

Classifiers	The result of 10-fold cross-validation on dataset 2		The result of using dataset 1 as a held-out dataset	
	Average of macro F1-scores	Standard deviation of macro F1-scores	Macro F1-score	Accuracy
K-Nearest Neighbors	0.61	0.02	0.56	0.70
SVM	0.76	0.07	0.63	0.93
Decision Tree	0.75	0.03	0.71	0.88
Random Forest	**0.79**	0.07	**0.80**	**0.94**
Ada Boost	0.68	0.07	0.62	0.92

The highest F1‐scores and Accuracy values.

As is shown in Table 7, the Random Forest classifier achieved the best results, and hence we proceeded to finetune this classifier. We observed that average F1 scores are reasonable for multiple classifiers (SVM, decision tree, and Random Forest); this highlights the fundamental feasibility of separating statements with claim from those without a claim.

The data that were used to train the final Random Forest classifier were the whole datasets 1 and 2 (1,126 records). Since dataset 2 was imbalanced and also it contained the majority of records, the whole training data became imbalanced. There were 594 records with “Negative” labels, 477 records with “Positive” labels, and only 55 records with “Unknown” labels. The data were extremely imbalanced since only 4.8 per cent of training data are annotated with “Unknown” labels. To tackle this, we generated synthetic examples via the Synthetic Minority Oversampling Technique (SMOTE), which generates new synthetic examples based on their selected nearest neighbors (Chawla et al., 2002).

To select the tunning parameters of the Random Forest classifier, GridSearchCV function of Scikit-learn (Pedregosa et al., 2011) was used. The tunning function was parameterized for training the Random Forest classifier with 5-fold cross-validation. The parameters that we tried to optimize were the numbers of estimators (n_estimators) and maximum depth (max_depth). The rest of the parameters used default values^4^. For the number of estimators, the range of [100, 150, 200, 250, and 300] and for the maximum depth, the range of [10, 20, 30, 40, and 50] were considered to find the best combination. The optimum Random Forest in terms of macro F1-measure corresponds to 200 estimators and a maximum depth of 40. In the final step, the Random Forest classifier was trained on the oversampled datasets 1 and 2. Table 8 illustrates the performance of the model assessed on the unseen test data, dataset 3.

**TABLE 8:** The performance of detecting claims on the test data (dataset 3) based on each class.

	Precision	Recall	F1-score	# of instances
Positive	**0.97**	0.91	**0.94**	102
Negative	0.95	**0.93**	**0.94**	99
Unknown	0.33	0.60	0.43	10

The highest Precision, Recall and F1-score values.

As you can see, the results are shown based on each class based on precision, recall, and F1-score. All the scores for positive and negative classes are more than 90 per cent. For precision, the positive class has the highest score; for recall, the negative class. In terms of F1-score, both categories achieve the same score. The unknown category was extremely imbalanced. Regarding the “Unknown” category that was extremely imbalanced, there were only 55 responses in training data, datasets 1 and 2, and only 10 in the test dataset 3. Overall, the performance on the positive and the negative class was very satisfactory. For the unknown class, it was not. The imbalanced precision and recall values mean that given an “unknown” label for a user statement, there is a reasonable likelihood that it will be wrongly classified as unknown (low precision). On the other hand, there is a very small likelihood of a user statement labeled as positive or negative to be anything else. In a separate experiment, a new model was created without using SMOTE, for the second time but as an up-sampling method. Without using SMOTE, all the scores of the minority class were zero.

Furthermore, since the claim detection model was a multi-class classifier, macro and weighted metrics are reported. Besides these scores, overall accuracy and Cohen’s κ are reported. In Table 9, the macro and the weighted score of precision, recall, and F1-score and also accuracy and Cohen’s κ are illustrated.

**TABLE 9:** The overall performance of detecting claims on the test data (dataset 3).

Random forest classifier	Precision	Recall	F1-score
Macro average	0.75	0.81	0.77
Weighted average	0.93	0.91	0.91
Accuracy	0.91		
Cohen’s κ	0.83		

Macro average precision, which is the average of precision of all classes, is 0.75. However, weighted average precision, which is the average precision based on the number of records for each class, is 0.93. We also measured macro and weighted average for recall and F1-score metrics. The claim model had 0.91 accuracy which is satisfiable.

#### 5.1.2 Warrant Component

In this subsection, we describe the development and evaluation of a classifier for deciding the existence of an explicit warrant in the sense of an explicit reference to one of five predefined different views on intelligence. In Table 10 several real responses from the study are shown.

**TABLE 10:** Real samples regarding the different values of the warrant component.

User’s response	Warrant
“Yes, I think that any action that involves the act of thinking and acting, involves a certain level of intelligence, in my opinion they are very intelligent, because they are born doing things that we humans are not born doing, they learn new things, things which is outside the animal world, things that only we humans learn, but of course there is a limitation in that.”	With
“I think a monkey is very intelligent because it can learn just like a human.”	With
“Snakes have the ability to adjust their behavior as determined by their surroundings and, as such, are able to learn from their experiences, so, yes, they are intelligent.”	With
“A self-driving car is intelligent as long as it has the correct information for it to function. It needs to have “brains” in order to work properly.”	Without
“No, I think that the actins of reptiles which include apparent stealth and self-direction, do not correspond to selecting from a set of alternative actions. The action is the only option and it is conjured by the needs of instinct”	Without
“It was intelligent it shows the friendship between two countries namely France and United States and mostly it representing liberty the enlightening the world. The torch really shows the path to freedom.”	Without

Again, we compared given standard machine learning classifiers (K-Nearest Neighbors, SVM, decision tree, Random Forest, and Ada Boost) similar to what we did for the claim component. The result is shown in Table 11.

**TABLE 11:** The result of 10-fold cross-validation on dataset 2 in detecting warrants and evaluation of performance on the held-out dataset.

Classifiers	The result of 10-fold cross-validation on dataset 2		The result of using dataset 1 as a held-out dataset	
	Average of F1-scores	Standard deviation of macro F1-scores	Macro F1-score	Accuracy
K-Nearest Neighbors	0.76	0.03	0.55	0.58
SVM	0.85	0.03	0.61	0.61
Decision Tree	0.81	0.04	0.64	0.64
Random Forest	**0.87**	0.02	**0.68**	**0.68**
Ada Boost	0.85	0.03	0.65	0.65

The highest F1‐scores and Accuracy values.

Based on the results in Table 11, Random Forest classifiers were selected for detecting the existence of warrant in user’s responses. Similar to the claim classifier, GridSearchCV function of Scikit-learn (Pedregosa et al., 2011) was used to tune the parameters of the Random Forest classifier. The hyperparameters that we tried to find their optimum values were the numbers of estimators (n_estimators) and maximum depth (max_depth). For the first hyperparameter, the number of estimators, the range of [50, 100, 150, 200, and 250] and for the maximum depth, the range of [10, 20, 30, 40, and 50] were considered to find the best combination. The optimum Random Forest in terms of F1-measure corresponds to 100 estimators and a maximum depth of 30. Table 12 reports the performance of the model assessed on the unseen test data, dataset 3.

**TABLE 12:** The overall performance of detecting warrants on the test data (dataset 3).

Random forest classifier	Precision	Recall	F1-score	# of instances
With warrant	0.95	0.83	0.88	111
Without warrant	0.83	0.95	0.88	100
Accuracy	0.89			
Cohen’s κ	0.77			

Based on Table 12, the category of “with warrant” had the highest precision; however, the best recall was related to “without warrant” category. The overall accuracy and Cohen’s κ were 0.89 and 0.77 respectively. Besides the metrics, which are reported in Table 12, the average F1-score for the model was 0.88. These values are overall very reasonable. Especially, however, we note that for our use case, the lower precision and higher recall for “without warrant” means, additional fine-tuning might need to penalize further a wrong “without warrant” classification; in this case the conversational agent would mistakenly ask for an explicit warrant (a reference to one of the five definitions of intelligence in our study) even though the user had already given one. This should only be done given substantial evidence that such a question does more harm (=annoys users) than good (=helps users develop clear argumentative structures).

#### 5.1.3 Evidence Component

In this subsection, we describe the development and evaluation of a classifier for deciding the existence of concrete evidence that illustrates the (non-)intelligence of an entity lasts. In Table 13, several responses are shown.

**TABLE 13:** Real samples regarding the different values of the evidence component.

User’s response	Evidence
“In my opinion, a monkey is an intelligent being, as he presents aspects similar to those in humans, such as concern for the group, being able to perceive what is best for his community with its due limitations, motor intelligence, intelligence to solve situations that demand creativity.”	With
“Actually, yes, I do. It doesn’t “think humanely, or act humanely.” I’m not sure if it thinks rationally or not, but it acts rationally: seeking out light in order to maximize its nutritional opportunities. It also, as all plants, learns from experience, in that it grows to match environmental conditions.”	With
“I don’t believe Google search engine meets the definition of intelligent because humans are behind the code of Google so Google itself is not doing the thinking. It is also only acting on what humans tell it to do. The only learning it might do is remembering what you’ve searched for previously and remembering cookies.”	With
“Based on the definition provided the venus fly trap is not intelligent. I believe it meets some of the criteria (Thinks and acts rationally, learns from experience) but not all. It does not think or act humanly”	Without
“yes because it behaves humanly and can be able to adapt to changes to its environment”	Without
“A Table is unintelligent, because it cannot think like a human, move on its own or adapt behavior to a changing environment.”	Without

Similar to the warrant and claim section, we compared given standard machine learning classifiers (K-Nearest Neighbors, SVM, decision tree, Random Forest, Ada Boost). The result is shown in Table 14.

**TABLE 14:** The result of 10-fold cross-validation on dataset 2 in detecting evidence and evaluation of performance on the held-out dataset.

Classifiers	The result of 10-fold cross-validation on dataset 2		The result of using dataset 1 as a held-out dataset	
	Average of F1-scores	Standard deviation of F1-scores	Macro F1-score	Accuracy
K-Nearest Neighbors	0.87	0.02	0.70	0.77
SVM	0.86	0.01	0.44	0.70
Decision Tree	0.86	0.01	**0.72**	0.77
Random Forest	**0.90**	0.01	**0.72**	**0.81**
Ada Boost	0.88	0.02	0.63	0.74

The highest F1‐scores and Accuracy values.

Similar to the claim and warrant classifiers, GridSearchCV was used for fine-tuning the model’s parameters by training on datasets 1 and 2 together. The tunning function was parameterized for training a Random Forest classifier in which 5-fold was selected for cross-validation with different numbers of estimators and maximum depth. The values that we considered for the number of estimators were [100, 200, 300, 400, and 500] and for the maximum depth were [40, 50, 60, and 70]. The optimum Random Forest in terms of the average of F1-measure corresponds to 300 estimators and a maximum depth of 60. After finding the best parameters, a Random Forest classifier was trained on datasets 1 and 2. The performance of the model was assessed on the unseen test dataset 3 (Table 15).

**TABLE 15:** The overall performance of detecting evidence on the test data (dataset 3).

Random forest classifier	Precision	Recall	F1-score	# of instances
With evidence	0.83	0.96	0.89	159
Without evidence	0.79	0.42	0.54	52
Accuracy	0.83			
Cohen’s κ	0.45			

Based on Table 15, the highest precision and recall were related to the category of “with evidence.” The overall accuracy and Cohen’s κ were 0.83 and 0.45 respectively. In addition to the metrics mentioned in Table 15, the average F-score for this model was 0.80. In our case, identifying the evidence component is the most challenging part in comparison with the other components, since the evidence part of an argument is based on users’ experiences or observations. These precision and recall values are overall very reasonable. In comparison with the warrant classifier, in which users needed to mention warrants explicitly, there was no explicit answer for the evidence part. Thus, even if the evidence classifier wrongly predicts the category of “without_evidence,” the whole conversation remains coherent because, in this case, the agent just asks the user to elaborate more the response.

### 5.2 Can a Conditional Dialogue Structure With Conditions Based on the Existence of Components From Toulmin’s Model of Argument Lead to Coherent Conversations in the Given Domain? (RQ3)

Above, we have ascertained that the existence of core components from Toulmin’s model of argument can be detected reasonably well for the given dataset. In this section, we ask whether the availability of such classifiers enables us to create a conditional dialogue structure that can lead to coherent conversations (RQ3). We answer this research question by example, in the following senses: First, we are just looking for a single reasonable dialogue structure. There surely are many reasonable dialogue structures, but we just need one. Also, in the current study, we are just interested in showing that the dialogue structure can lead to coherent conversations; not in showing how often this is the case in a given setting. In showing the quality of the conditional dialogue structure we, therefore, use the concept of conversation coherence as a quality indicator. By coherence we understand the linguistic concept of coherence, as denoting the extent to which a text (in this case: the conversation between the agent and the user) is meaningful and thoughts in it are well and logically connected. We, therefore, use conversational coherence as a fundamental quality that a tutorial conversation needs to have.

Coherence in the case of a retrieval-based conversational agent relies on the quality of 1) the conditional dialogue structure and 2) the developed classifiers as well as the alignment of the two. The conditional dialogue structure needs to be well designed in that it is overall a reasonable path through a conversation, with an introduction, and a reasonable sequence of questions that suit the overall goal. The developed classifiers need to be able to decide between the conditional branches. The alignment between the two is necessary because depending on the quality of the classifiers, the responses of the agent need to show a different level of confidence toward the human user, in order to better perform in cases of the wrong classification.

In the below example conditional dialogue structure, the question about an entity’s intelligence is shown as embedded in a longer tutorial interaction. The interaction follows the revised version (Anderson et al., 2001) of Bloom and others, (1956)’s proposed taxonomy of educational goals. In the revised version, the first four steps of the taxonomy are introducing knowledge, remembering, understanding, and applying the knowledge. The focus of this article was on the applying step. Here we elaborated on each step.

**FIGURE 2:**
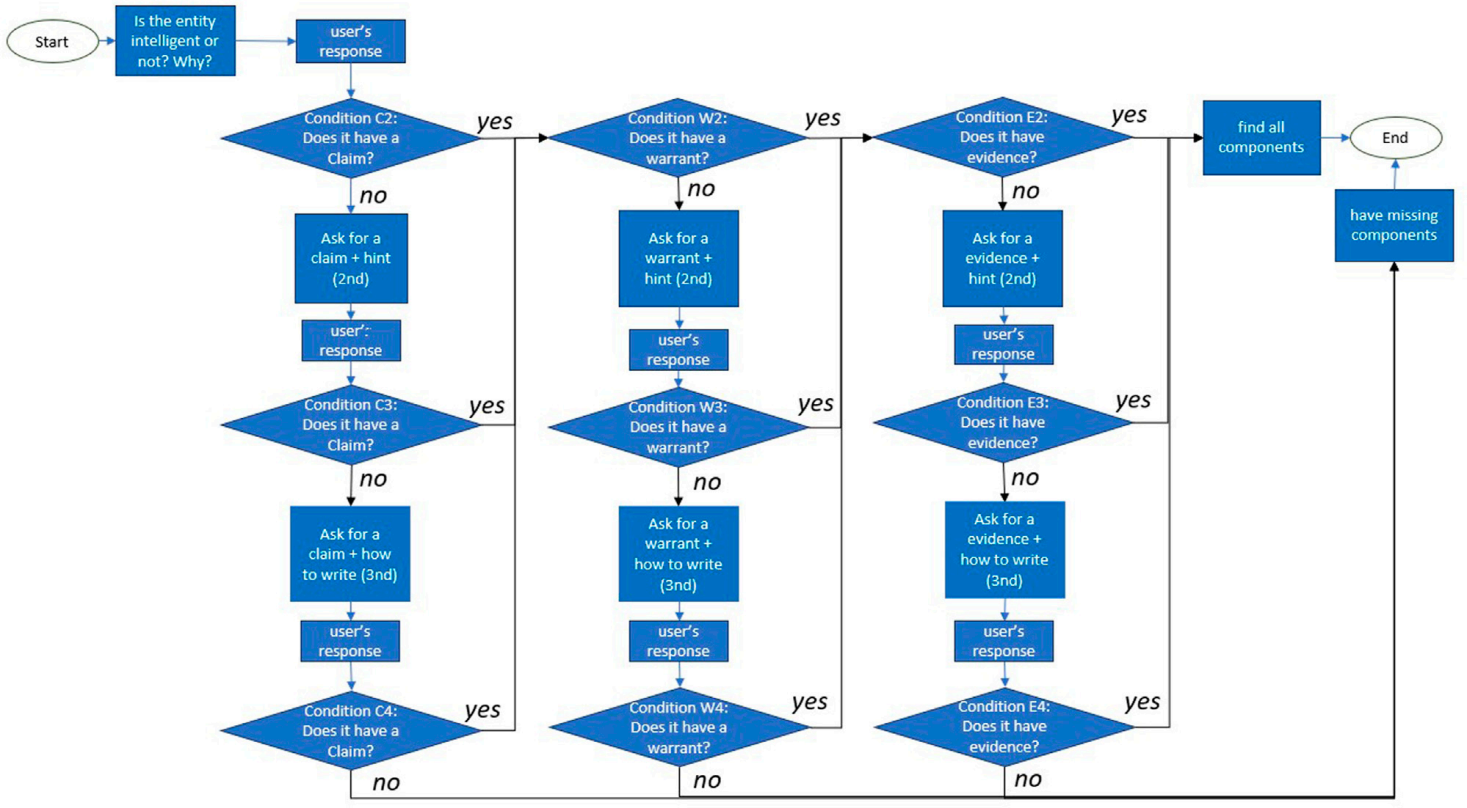
The different states that the agent reaches based on the user’s responses regarding the main question of the conversation, “*Is < an entity > intelligent or not? Why?*”

• Introduction: The introduction is adapted to a use case setting in which the definitions of intelligence have already previously been discussed, e.g., in an introductory lecture on artificial intelligence.
• Remember: This part asks the user to repeat the learned definitions. Conditional branching with feedback can be designed for, but was outside our scope in this article.
• Understand: This part asks the user to explain in own words. Conditional branching with feedback can be designed for, but was outside our scope in this article. Additionally, it could be advisable even if problematic reasoning were detected here, to proceed immediately to the application stage, in order to switch between concrete and abstract reasoning; and only to come back to this level of understanding after a successful argumentation on a concrete example was carried out.
• Apply: This part is in focus of the present article, and the goal of the below dialogue structure is to show how the classifiers that decide upon the existence of core components of Toulmin’s model of argument can be used to decide between branches in the dialogue structure. The dialogue flowchart is illustrated in Figure 2; in the subsequent explanation, the identifiers in brackets denote the decision points from Figure 2. The classifiers are executed sequentially. We first check for the existence and direction of a claim (C2), and act on identifying a missing claim; then we check for the existence of a warrant (W2), and act on identifying a missing warrant; finally, we check for the existence of evidence (E2), and act on identifying a missing evidence. Whenever a component (claim, warrant, evidence), is detected as missing, the agent uses increasing levels of scaffolding. The first scaffold is to point concretely to the missing core component (C3, W3, and E3); the second scaffold is to give the learner the start of an argumentative sentence or paragraph that just needs to be completed (C4, W4, and E4). When the last scaffold fails, in the current dialogue structure, the conversation is (gracefully) ended, currently by apologizing for its own capability.

**FIGURE 3:**
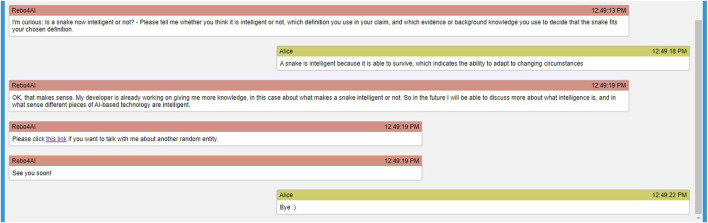
A coherent conversation when all the core components were mentioned by the user.

**FIGURE 4:**
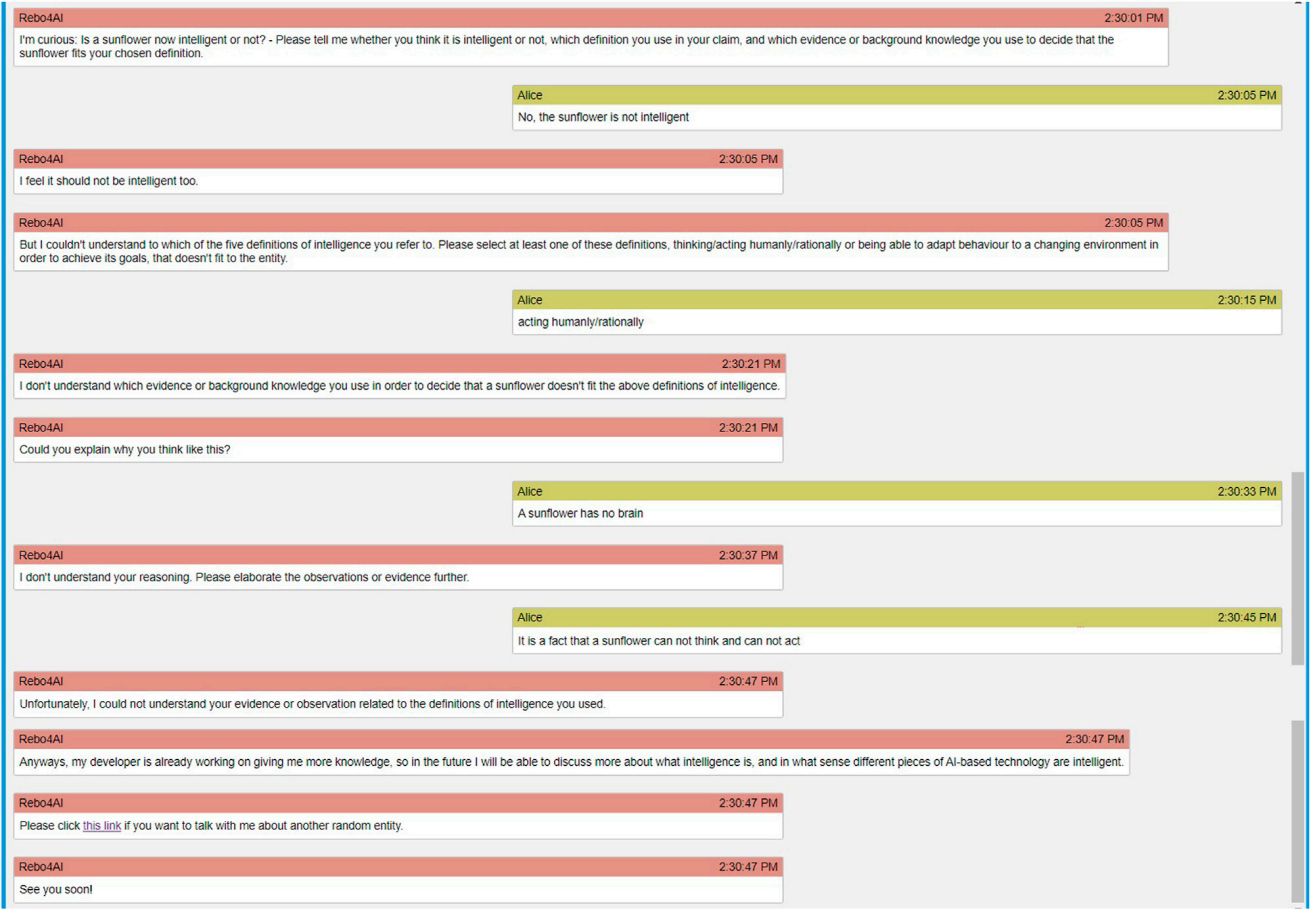
A coherent conversation when some of the core components were not mentioned by the user.

In Figures 3, 4, two example conversations are given that showcase coherent conversations. In the conversation shown in Figure 3, the agent asks the user to argue whether and why a snake is intelligent. The user’s response, “*A snake is intelligent because it is able to survive, which indicates the ability to adapt to changing circumstances*” (a response from dataset 2), passes through all three classifiers (claim, warrant, evidence, see *How Well Can Components of Toulmin’s Model of Argument Be Identified in the Given Domain? (RQ2)*). Subsequently, the tutorial conversation is over. In the conversation shown in Figure 4, the agent asks the user to argue whether and why a sunflower is intelligent. the user’s response, “*No, the sunflower is not intelligent*,” only contains the claim, but no warrant or evidence. In this case, the agent first shows its agreement and then asks for warrants to complete the argument. From this step onward, it was us as authors who completed the remainder of the tutorial dialogue, just to show how a reasonable dialogue could ensue: The user’s utterance, “*acting humanly/rationally*,” fulfills the lack of the warrant component. Now, the only missing component is the evidence. In this step, the agent requests the user to add evidence or background knowledge to justify the claim. If the agent cannot identify the missing components, it will give the second chance to the user and ask again. As the agent again cannot find the evidence component, it asks the user to elaborate again. This is the last chance and if the agent cannot find the missing components again, the conversation will be ended. In Figure 4, the agent needed to find a connection between having no brain and thinking or acting to consider it as evidence part.

The agent’s responses and follow-up questions are selected based on the predictions of classifiers. So the conversations are coherent if the classifiers perform correctly. If they do not, the conversations can still be coherent as shown in the second example. This is created by the agent showing uncertainty when asking the user (=learner) to elaborate (C4, W4, and E4)^5^.

The above example conversations show that coherence can be achieved with the example conditional dialogue structure that makes use of classifiers that identify the existence of claims, warrants, and evidence as core components of Toulmin’s model of argument.

Note that the example conditional dialogue structure does not show how incoherent user responses can be caught and reacted to; and appropriate responses to wrong answers for the stages of remembering and understanding are not discussed either in this article.

### 5.3 Can Toulmin’s Model of Argument be Used to Model Different Types of Structural Wrongness Within Conversational Agents in the Given Domain? (RQ1)

In this subsection, we respond to the overarching research question, whether and how Toulmin’s model of argument is a suitable basis for modeling different types of the wrongness of arguments for use within conversational agents (RQ1). Answering this research question will also immediately lead over to a broader discussion of our work in *Discussion and Conclusion*.

First, we point out that using Toulmin’s model of argument allows us to assess structural characteristics of responses and in this sense structural quality. This means that we can detect the existence of components of a reasonable argument, but we cannot—not by using Toulmin’s model of arguments—say anything more about the content-wise plausibility.

For this purpose, we find that Toulmin’s model of arguments works very well: With a comparatively small dataset, we were able to develop reasonably accurate classifiers (see *How Well Can Components of Toulmin’s Model of Argument Be Identified in the Given Domain? (RQ2)*) that are useful within a conditional dialogue structure to decide between branches (RQ2). The developed classifiers identify the existence of necessary components (the claim, warrant and evidence). Even though the classifiers model structural quality of users' messages, this assessment is also related to content. The “warrant” classifier uses as features substantially content of the pre-defined definitions. The “evidence” classifiers use as features substantially content-related keywords that relate to how people argue about the intelligence or non-intelligence of entities. This highlights that in quality, structure and content are inter-related.

Despite this dependence of assessing structural quality on content-related features (∼quality indicators), we secondly observe that identifying the existence of Toulmin’s core argumentative components does not per se allow us to assess content-wise plausibility of the made argument. For instance, in this response “*yes because it acts rationally by providing humans comfort*,” in which *it* refers to an office chair, all the core components of Toulmin’s model of argument were mentioned, but the content is arguable. However, we could use Toulmin’s argument components to model different types of wrongness: For instance, it could be that the evidence per se is not correct (a fictional example could be to say that “snakes are regularly observed to talk with each other in sign language”); it could also be, however, that the given evidence does not usefully relate to the used definition (“sunflowers move their heads with the direction of the Sun, which shows that they learn from experience”). More generally speaking, each of Toulmin’s model of arguments can have an independent value of “correctness” (whereby the value, in general, cannot be assumed to be binary), as well as interconnected values of content-wise quality in terms of how well, content-wise, the different parts of the argument align with each other.

Following this observation, we ask, how such content-wise quality assessment can be implemented? The answer to this can be found both in existing literature, and in future work: In the existing literature on argument mining, both the identification of similar arguments to one made by the user, and the identification of groups of arguments have been treated (Adamson et al., 2014). Argument similarity can be used when expert statements are available in the sense of a gold standard, and grouping arguments can be used when agreement with a majority opinion is a good marker of argument quality. On the other hand, fine-granular argument component detection and reasonable links between components that on their own might be correct or at least sufficiently reasonable to identify further problems are a topic for future research.

## 6 Discussion and Conclusion

In summary, our work indicates that Toulmin’s model of arguments provides a useful conceptual structure on which to base classifiers that help a conversational agent decide between different branches in a conversation that supports learning.

In the present article, we have shown this for a particular conversation around in what sense a given entity is regarded as intelligent or not. We have shown this based on a dataset of answers to this question that has been collected outside of a conversational agent. Furthermore, we used our results to show a dialogue structure for a conversational agent based on the developed classifiers.

Our work also has several limitations: First, in our data collection task, study participants received a specific explanation of what constitutes a good argument. Our concern was to have sufficient numbers of arguments that contain all components of Toulmin’s model. Furthermore, on the background of our research being on educational technology, it is reasonable to expect that users would receive some explanation for this. However, in settings, where no a priori explanation is given, it is to be expected that the distribution of classes (which components of Toulmin’s model exist in a given user statement) is different than the distribution in our data set; and subsequently performance of the developed classifiers will vary.

Second, we have shown these results for a particular conversation. The classifiers use domain-specific (i.e., dataset-specific) features, like the limited-length TFIDF vectors, or the thirty terms most highly correlated with the “evidence” label (see *Feature Selection*). This means, for different conversation topics, still some feature reengineering would need to occur. While our *approach* to feature engineering can be assumed to generalize, this is 1) an assumption and 2) still will result in different concrete features. Examples of a different conversation that is structurally similar to the one discussed in this article are an ethical dilemma. By definition, ethical dilemmas are situations in which, depending on underlying prioritization of different obligations, different courses of action would be reasonable. Such conversations could be conceptualized in Toulmin’s model of argument as laying out as a claim which course of action one would choose (claim), laying out which obligation was most highly prioritized in choosing this course of action (warrant), and giving additional reasoning as to why the chosen priority is reasonable (evidence).

Third, as discussed above in *Can Toulmin’s Model Of Argument Be Used To Model Different Types Of Structural Wrongness Within Conversational Agents In The Given Domain? (RQ1)*, while we do argue that Toulmin’s model of arguments can also be used to structure identifying content-wise types of wrongness in arguments by means of argument mining, we have not shown this in the present work. Finally, we have not shown the effect of conversing with the agent on actual learning in an experimental study with human subjects.

These limitations also point out the direction of interesting future work, and stand in for research challenges that are being widely understood to be ambitious and are being addressed in educational technology and conversational agent research at large: Transferability of domain-specific classifiers; identifying more complex types of wrongness in arguments (i.e. argumentations where single components may make sense but do not fit together, as discussed toward the end of *Can Toulmin’s Model Of Argument Be Used To Model Different Types Of Structural Wrongness Within Conversational Agents In The Given Domain? (RQ1)*) and effectiveness of conversational agents as intelligent tutors in comparison with other teaching methods.

Knowing these limitations, the contributions that this article makes toward state-of-the-art are 1) to give evidence that reasonably accurate classifiers can be built for the existence of single components of Toulmin’s model of arguments in (short) argumentative statements as would be expected in the context of a conversation with an intelligent agent in a given domain, 2) to show by an argument that such classifiers are useful within dialogue structures of conversational agents that are designed based on Bloom’s taxonomy for learning, and 3) to show by argument how the same conceptual structure of Toulmin’s model of argument can be used to further structure the identification of more complex types of faulty argumentation. These contributions complement existing research that has worked on longer argumentative essays (Wambsganss et al., 2020), which has differently conceptualized argumentation quality that is however less suitable for direct feedback within a conversational agent, and broader work on argumentation mining on identifying groups of similar arguments (Wachsmuth et al., 2017b) or conversational agents for factual teaching (Ruan et al., 2019).

## Data Availability

The original contributions presented in the study are included in the article/Supplementary Material, further inquiries can be directed to the corresponding author.

## Footnotes

1
https://www.cs.cornell.edu/people/tj/svm_light/svm_hmm.html

2
https://www.mturk.com/

3 The data have been uploaded as Supplementary Material

4 The version of 0.23.2 of Scikit-learn was used in this study

5 All agent’s responses and branches are listed in a table (Supplementary Table S1) in the supplementary materials
