# Supplementary material for: Developing a Conversational Agent’s Capability to Identify Structural Wrongness in Arguments Based on Toulmin’s Model of Arguments
Source: Front Artif Intell. 2021 Nov 30;4:645516. doi: 10.3389/frai.2021.645516 (PMC8680349; doi:10.3389/frai.2021.645516)

Supplementary Material

1 Supplementary Data

The datasets that collected for this study are uploaded as a separate zip file, named “dataset.zip”. This dataset was used to create classifiers by which we could identify the existence of the core components of argument based on Toulmin’s model of argument. The data collected by Amazon Mturk and annotated based on the core components (claims, warrants, and evidence). The more details are mentioned in ReadMe.txt file in dataset.zip.

2 Chatbot’s URL

By clicking here, you can talk to Rebo4AI, the agent that mentioned in the paper. Since we have been working on it, you may have different experience in comparison to what mentioned in the paper. Please contact the corresponding author if the chatbot’s webpage is down.

3 Supplementary Figures and Tables

In Supplementary Table 1, all the steps and conditions that our agent reacts based on are illustrated.

Supplementary Table 1. The whole dialog structure and branches.

Hi, I’m Rebo4AI, a prototypical conversational agent. With me you can currently discuss only about intelligence. Is that OK with you; do you want to start a conversation? (please answer “yes” to continue) … (continues of a positive answer is given, actually a few more answers than yes are accepted)	**Introduction**
Do you remember the definitions given in XXX – what are they?	**Remember** definitions of intelligence (this part would need to be changed depending on the educational context in which this agent is embedded)
What do you think, what does <entity – chosen randomly from the list of definitions> actually mean?	**Understand** definitions of intelligence
So, I’m curious: Is <an entity> now intelligent or not? Please tell me whether you think it is intelligent or not, which definition you use to make this claim, and which evidence of background knowledge makes you think so?	**Apply** definitions of intelligence to an example. This is the first chance for users to mention claims, warrants and evidence.
**Claim conditions: Looking for the missing claim component**	
Mmmm, I don't get your opinion about it. Could you tell me clearly whether it is intelligent or not? And then explain why you think like that?	**Condition C2**: This is the second try for getting the user’s claim. The concretely missing core component (claim) is pointed out.
Unfortunately, I cannot understand what you just said. Please try to answer the original question of whether <an entity> is intelligent or not, and why, by starting the argumentation with: "The entity is (not) intelligent because …"	**Condition C3**: This is the third and last try for getting the user’s claim. A sentence starter is given to scaffold the correct argument.

<table>
<tr><td>I could not understand your assertion regarding the entity. I think I need to improve my understanding to find out what you are saying. So, let's talk later.</td><td>Condition C4: After the third chance, the agent will end the conversation, if until here we haven’t been able to understand the claim, either the user isn’t engaging with the agent, or the classifiers are substantially failing.</td></tr>
<tr><td colspan="2">Warrant conditions: Looking for the missing warrant component</td></tr>
<tr><td>I feel it should be intelligent too.
Interesting! you think it is intelligent.
I feel it should not be intelligent too.
Interesting! you think it isn’t intelligent.</td><td rowspan="2">Condition W2: Based on the user’s claim and the entity, the agent gives different feedback. The first line is actually adaptive to the direction of the user’s claim and whether this agrees with what is laid down as the direction of the claim that was defined as most reasonable by the authors. This is the second try for getting the user’s warrant.</td></tr>
<tr><td>But I couldn't understand to which of the five definitions of intelligence you refer to. Please use at least one of these definitions explicitly when arguing why <an entity> is (not) intelligent: thinking/acting humanly/rationally or being able to adapt behavior to a changing environment in order to achieve its goals.</td></tr>
<tr><td>Mmmm, I’m sorry, I still don’t understand which of the five definitions you refer to.

Could you please try and phrase your answer like this: <the entity> is (not) intelligent in the sense of (not) being able to act or think rationally." You should mention some of the definitions and then explain why they (don’t) fit to the entity.</td><td>Condition W3: This is the third try for getting the user’s warrant. Based on the user’s claim, the agent tries to help the user by saying how to write. A sentence starter is given to scaffold the correct argument. The second part of the response will change based on the user’s claim.</td></tr>
<tr><td>Unfortunately, I could not understand based on which definitions you said the entity is intelligent.

Anyway, my developer is already working on giving me more knowledge, so in the future I will be able to discuss more about what intelligence is, and in what sense different pieces of AI-based technology are intelligent.</td><td>Condition W4: After the third chance, the agent will end the conversation.</td></tr>
<tr><td colspan="2">Condition 3: Looking for the missing evidence component</td></tr>
<tr><td>Great, I think I understand already a lot of what you’re saying – one thing isn’t clear yet: I don't understand which evidence or background knowledge you use in order to decide that <the entity> fits to the definition of intelligence you used above.

Could you explain why you think like this? You can talk about the characteristics of <the entity> to show that how <the entity> (doesn’t) fits to the definitions.</td><td>Condition E2: This is the second try for getting the user’s evidence. The second part of the response will be changed based on the user’s claim.</td></tr>
<tr><td>Mmmm, I’m sorry, I still don’t understand how you argue that <the entity> (doesn’t) fits to the definitions.</td><td rowspan="2">Condition E3: This is the third try for getting the user’s warrant. Based on the user’s claim, the explanation about the evidence will be changed.</td></tr>
<tr><td>For instance, you can say monkey can act rationally because they know how to take care of themselves. Or they can make tools which is related to learn new</td></tr>
</table>

things to achieve goals. So, how do you justify the mentioned definitions fit to <the entity>?<br><br>For instance, you can say since a pen is an inanimate object, it cannot act or think. That is why a pen cannot be called intelligent. So, how do you justify the mentioned definitions fit to <the entity>?	
Unfortunately, I could not understand your evidence or observation related to the definitions of intelligence you used.<br>Anyways, my developer is already working on giving me more knowledge, so in the future I will be able to discuss more about what intelligence is, and in what sense different pieces of AI-based technology are intelligent.	**Condition E4**: This is the last chance of the user to talk about his or her observation or experience regarding his or her claim. After the third chance, the agent will end the conversation
**All the core components are mentioned.**	
OK, that makes sense. My developer is already working on giving me more knowledge, in this case about what makes <the entity> intelligent or not. So, in the future I will be able to discuss more about what intelligence is, and in what sense different pieces of AI-based technology are intelligent.	This is the agent response when all the core components were mentioned by the user.

In Supplementary Figure 1, 2 and 3 are related to Section 5 of the paper in which the results are illustrated. Supplementary Figure 1 shows the different values of F1-score based on the different combination of two hyperparameters (n_estimators, max_depth) in detecting claim component. Supplementary Figure 2 illustrates the F1-score values which obtained during the tuning hyperparameters for identifying warrant. And in the last figure, Supplementary Figure 3, the performance (based of F1-score) of the evidence detecting model for different values of n_estimators and max_depth is shown.

Supplementary Figure 1. The different F1_macro values that obtained during tuning

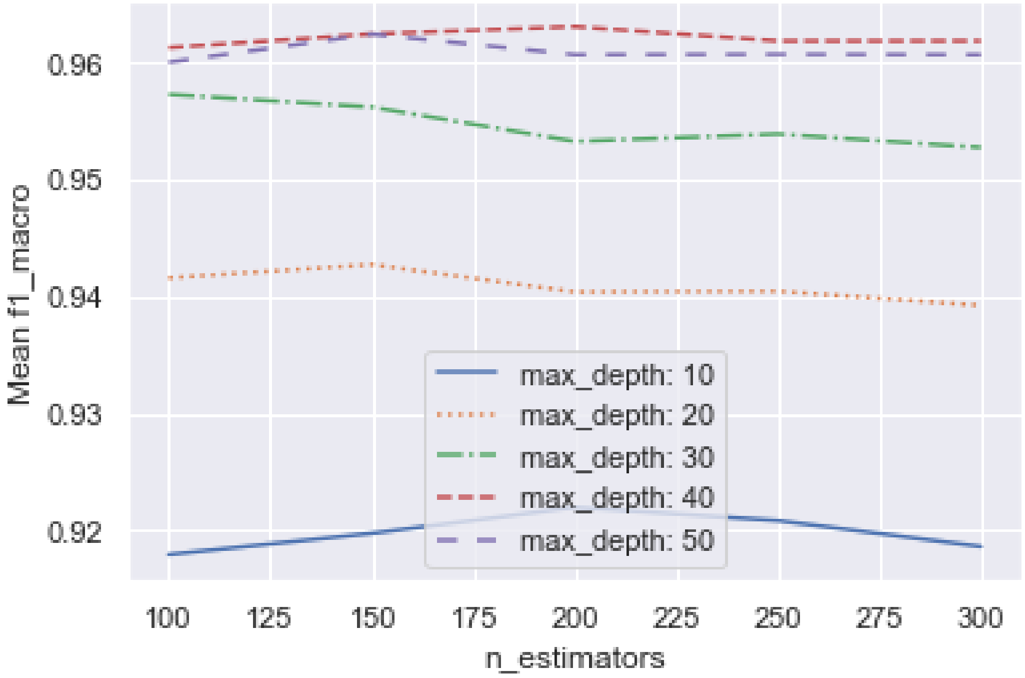

hyperparameters in detecting the claim component.

Supplementary Figure 2. The different F1_macro values that obtained during tuning hyperparameters in detecting the warrant component.

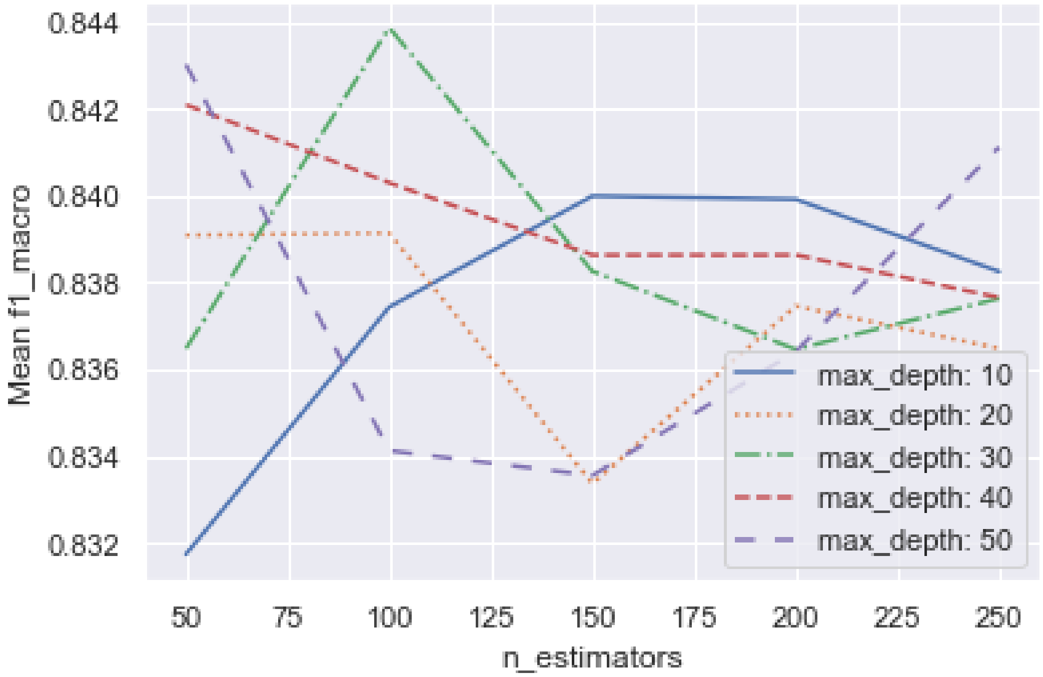

Supplementary Figure 3. The different F1_macro values that obtained during tuning hyperparameters in detecting the evidence component.

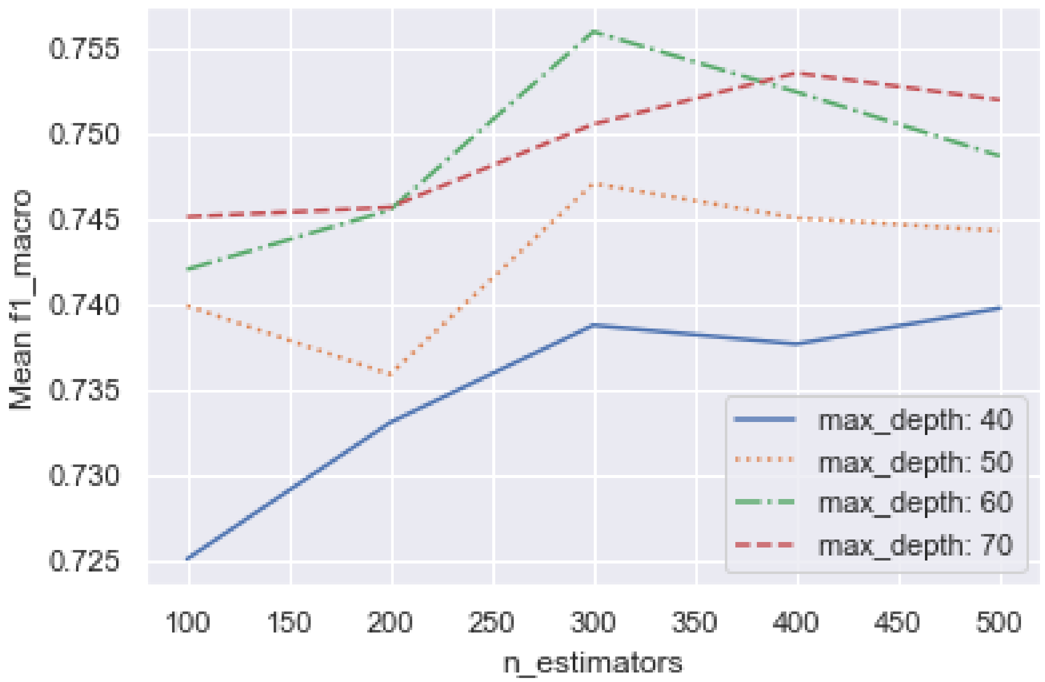

Supplement: Supplementary file 1 [file DataSheet1.zip › Supplementary Material.pdf]
